# Overview of clinical forensic services in various countries of the European Union

**DOI:** 10.1080/20961790.2019.1656881

**Published:** 2019-10-04

**Authors:** Sophie Kerbacher, Michael Pfeifer, Reingard Riener-Hofer, Andrea Berzlanovich, Maeve Eogan, Anita Galić Mihic, Gregor Haring, Petr Hejna, Johannes Höller, Sorin Hostiuc, Michael Klintschar, Peter Kováč, Astrid Krauskopf, Simone Leski, Michal Malacka, Thorsten Schwark, Hanna Sprenger, Andrea Verzeletti, Duarte Nuno Vieira, Sylvia Wolf, Kathrin Yen

**Affiliations:** a Ludwig Boltzmann Institute for Clinical Forensic Imaging, Ludwig Boltzmann Gesellschaft, Graz, Austria;; b Center of Forensic Medicine, Medical University of Vienna, Vienna, Austria;; c Department of Obstetrics and Gynaecology, Rotunda Hospital, Dublin, Ireland;; d Institute of Forensic Medicine and Criminalistics, School of Medicine, University of Zagreb, Zagreb, Croatia;; e Department for Forensic Medicine and Deontology, University of Ljubljana, Ljubljana, Slovenia;; f Department of Forensic Medicine, Charles University and University Hospital, Hradec Kralove, Czech Republic;; g Department of Legal Medicine, National Institute of Legal Medicine, Bucharest, Romania;; h Department for Legal Medicine, Hannover Medical School, Hannover, Germany;; i Forensic.sk, Inštitút Forenzných Medicínskych Expertíz, Bratislava, Slovakia;; j Institute of Forensic and Traffic Medicine, University of Heidelberg, Heidelberg, Germany;; k Faculty of Law, Palacký University Olomouc, Olomouc, Czech Republic;; l Department of Forensic Medicine, Laboratoire National de Santé, Dudelange, Luxembourg;; m Department of Medical and Surgical Specialties, Radiological Sciences and Public Health, University of Brescia, Brescia, Italy;; n Department of Forensic Medicine, Ethics and Medical Law, Faculty of Medicine, University of Coimbra, Coimbra, Portugal

**Keywords:** Forensic sciences, clinical forensic services, violence, JUST_e_U!, Directive 2012/29/EU, victim, examination

## Abstract

Examination of a person who has been a victim of a physical or sexual assault may be very important for upcoming legal proceedings. In the context of a clinical forensic examination, physical findings are recorded and biological trace material is gathered and secured. Ideally, all forensic findings are documented in a detailed report combined with photographic documentation, which employs a forensic scale to depict the size of the injuries. However, the integrity of such forensic findings depends particularly on two factors. First, the examination needs to be conducted professionally to ensure that the findings are properly admissible as court evidence. Second, the examination should take place as soon as possible because the opportunity to successfully secure biological samples declines rapidly with time. Access to low-threshold clinical forensic examinations is not evenly provided in all member states of the European Union (EU); in some states, they are not available at all. As part of the JUST_e_U! (**Ju**ridical **st**andards for clinical forensic examinations of victims of violence in **Eu**rope) project, the Ludwig Boltzmann Institute for Clinical Forensic Imaging in Graz, Austria created (in cooperation with its international partner consortium) a questionnaire: the purpose was to collect information about support for victims of physical and/or sexual assault in obtaining a low-threshold clinical forensic examination in various countries of the EU. Our paper provides a summary of the responses and an overview of the current situation concerning provided clinical forensic services.

## Introduction

In November 2016, the European Commission published a Special Eurobarometer Report on gender-based violence [1]: the aim was to assess the perception of citizens in the 28 member states of the European Union (EU) on the topic. In the report, gender-based violence is defined as “violence directed towards a person on the basis of their gender, and violence that disproportionately affects persons of a particular gender”; it therefore encompasses physical, sexual and psychological abuse. Any person, regardless of gender, can become a victim of gender-based violence, but women are particularly affected by this kind of violence [1,p.3]. In 2014, a survey by the EU Agency for Fundamental Rights (FRA) about violence against women found that one in three women in the EU older than 15 years had suffered from physical or sexual violence. The survey concluded that, “violence against women is … an extensive and wide-ranging fundamental rights abuse.” Moreover, the survey determined that the majority of women never report violence to the police or a victim-support organization. Therefore, such cases of violence may not appear in official criminal justice data, which results in a general lack of comprehensive data. The FRA recommends that health-care professionals should play an important role in countering the under-reporting of violence, encouraging victims to come forward, report their experience, and seek help. Health-care professionals need to be alerted about identifying violence and be able to recognize such cases. Hence, a questioning routine for health-care practitioners should be developed and include appropriate checks to clarify suspected abuse. If a patient has characteristic injuries that may have resulted from violence, 87% of questioned women indicated that they would consider it acceptable for an examining doctor to ask routinely about violence [2,p.7,11,15].

For the Special Eurobarometer Report on gender-based violence (2016), face-to-face interviews with over 27 000 EU citizens were conducted [1]. Over three-quarters of the respondents believed that domestic violence against women was common in their country; fewer than one-third considered that in their country, domestic violence against men was common. More than 90% of the respondents declared that domestic violence was unacceptable—either against women or men. In both cases, a large majority (around 80%) believed that the perpetrators should be punished by law. Regarding personal experience, almost one-quarter stated that they knew a family member or friend who was a victim; 70% of the respondents, who personally knew a victim, had talked to someone about the violent event; however, only one in ten disclosed the matter to the police, 8% spoke to health-care professionals, and only 7% contacted support services. As to the main reasons for 30% of the respondents not talking to anyone about the violent event, the survey found the following: they believed that it was none of their business; they lacked proof; they did not want to create trouble; or they stayed silent for no particular reason. The survey concluded that most cases concerning domestic violence affected women.

To tackle this issue, the Special Eurobarometer Report recommends further action against gender-based violence in the EU. Among other measures, it states that the Council of Europe Convention on preventing and combating violence against women and domestic violence, referred to as the Istanbul Convention, should be implemented by the member states [1,p.2,6,8,10,12–15,33,34].

## Legal regulations in connection with gender-based violence

In the preamble to the Istanbul Convention, it is stated as fact that women and girls are at greater risk of falling victim to gender-based violence than men. Article 2 Section 2 of the convention particularly recommends tackling gender-based violence. One provision is laid down in Article 25 concerning support for victims of sexual violence: it states that countries should take responsibility to “set … up … appropriate, easily accessible rape crisis or sexual violence referral centres for victims in sufficient numbers to provide for medical and forensic examination, trauma support and counselling for victims” [3]. The Explanatory Report to the Istanbul Convention specifies that these sexual violence referral centres can be specialized, for example in high-quality forensic practice (Recital 141). Moreover, the report emphasizes the “good practice to carry out forensic examinations regardless of whether the matter will be reported to the police, and to offer the possibility of having samples taken and stored so that the decision as to whether or not to report the rape can be taken at a later date” [4,p.26]. Accordingly, clinical forensic services should not be dependent on the victim making a formal complaint to the police regarding a criminal offence: a low-threshold access to clinical forensic examinations should be ensured [5].

The European legislator addresses gender-based violence in its “Directive 2012/29/EU of the European Parliament and of the Council of 25 October 2012 establishing minimum standards on the rights, support and protection of victims of crime” (ABl L 315, 57)—the so-called victims’ rights directive. In this context, Recital 17 states, “Women victims of gender-based violence and their children often require special support and protection because of the high risk of secondary and repeat victimization, of intimidation and of retaliation connected with such violence.” In particular, Articles 8 and 9 of the directive are dedicated to victim support services. Article 8 (“Right to access victim support services”) stipulates in Section 1 that member states have to provide “access to confidential victim support services, free of charge”. Article 9 (“Support from victim support services”) details in Section 1 the services to be offered as a minimum standard for victim support [6].

In advising European member states about implementation of the victims’ rights directive, the Directorate-General (DG) Justice Guidance Document regards Article 8 as one of the core rights of that directive. The document emphasizes that victim support plays a large role in helping victims in the process of their recovery. Support offers should be confidential, free of charge, available “from the earliest possible moment after a crime has been committed”, and irrespective of whether the crime has been reported. This is due to the fact that access to support services at an early stage can lower long-term consequences, such as suffering and loss of income. In addition, the DG Justice Guidance Document advises that the specific needs of a victim should be determined. For example, to process the circumstances of the crime, victims of sexual violence and domestic violence may require psychological support. Additionally, reliable support services may encourage a victim to make a formal complaint regarding the crime [7,p.24–26].

Thus, Article 8 Section 3 of the victims’ rights directive obliges member states to “establish free of charge and confidential specialist support services”; Article 9 Section 3 specifies which special services should be provided as a minimum. According to Article 9 Section 3 Littera b, victims with specific needs are “victims of sexual violence, victims of gender-based violence and victims of violence in close relationships”. Recital 38 recommends that among other victims, victims of gender-based violence should have special support services at their disposal as “immediate medical support, referral to medical and forensic examination for evidence in cases of rape or sexual assault” [6]. This is particularly important given that physical and sexual violence often goes unreported. Statistics reflect only reported cases of violence and so may just indicate the tip of the iceberg. There is also a link between citizens’ perception of domestic violence and their behaviour regarding formal complaints to the police: women in European states where domestic violence is considered less unacceptable “are less likely to report such violence” [8,p.13].

In consideration of all these matters, the Ludwig Boltzmann Institute for Clinical Forensic Imaging in Graz, Austria initiated the international JUST_e_U! (**Ju**ridical **st**andards for clinical forensic examinations of victims of violence in **Eu**rope) project [9].

## JUST_e_U! project

The European Commission provides financial contributions in the form of grants to projects, which help implement EU programmes or policies. To apply for grant funding, a project proposal has to be submitted under a specific call for proposals. In the case of the JUST_e_U! project, it was the Joint Justice & Daphne call – Actions grants to support national or transnational projects to enhance the rights of victims of crime/victims of violence (JUST/2015/SPOB/AG/VIC). The project was awarded a grant and co-funded by the Justice Programme of the EU [10]. The JUST_e_U! project started in February 2017 for a 2-year period: it addressed access to specialist support services, especially for clinical forensic examinations (Article 9 Section 3 Littera b in combination with Recital 38 of the victims’ rights directive) [5]. The project sought to reinforce the legal position of victims of sexual and/or physical violence: during a clinical forensic examination, injuries are documented in detail on a documentation form as well as with a camera and a forensic scale; trace evidence is collected and stored. These evidentiary findings can then be used in future legal proceedings [11].

To enhance victim support in this field on a European level, the project consortium involved the following: the Institute of Forensic and Traffic Medicine at the University Hospital Heidelberg [12] and Institute for Forensic Medicine at the Hannover Medical School [13] (Germany); the Department of Medical and Surgical Specialties, Radiological Sciences, and Public Health at the Università degli Studi di Brescia [14] (Italy); and the Department of Forensic Medicine at the Faculty of Medicine in Hradec Králové [15] and Faculty of Law at Palacký University Olomouc [16] (Czech Republic). The Ludwig Boltzmann Institute for Clinical Forensic Imaging [17] was the project leader.

One main part of the project focussed on dissemination and awareness-raising activities to expand understanding (among the public as well as among experts) of the importance of access to clinical forensic examinations for victim support. Accordingly, a project website [9] was established, and national symposia were hosted in each project partner country. Further, a 2-day workshop [18] for experts in clinical forensic medicine was organized by the Ludwig Boltzmann Institute for Clinical Forensic Imaging in early June 2018 in Graz. At that JUST_e_U! workshop, all project partners participated, and each recruited one additional forensic expert. In that way, it was possible to gather forensic expertize from 11 European countries: Austria, Croatia, Czech Republic, Germany, Ireland, Italy, Luxembourg, Portugal, Romania, Slovakia and Slovenia. The goals of the JUST_e_U! workshop were to discuss a future Clinical Forensic Network for Europe (CFN Europe) and a European-wide minimum standard for clinical forensic examinations.

To assess the starting point for the discussions, the Ludwig Boltzmann Institute for Clinical Forensic Imaging created in advance (in cooperation with its international partner consortium) two questionnaires. One questionnaire was dedicated to analyze the legal framework concerning clinical forensic examinations: Questionnaire concerning the legal framework for doctors when dealing with a case of physical violence. The results of the survey were analyzed by the project partner from the Faculty of Law at Palacký University Olomouc; they were summarized as a legal opinion, which was forwarded as a part of a compilation to European decision makers at the end of the project. Through the second survey, questions concerning the availability of clinical forensic service offers were addressed: Questionnaire concerning national victim supporting low-threshold clinical forensic examination offers (QCFN). Both questionnaires were drafted by the Ludwig Boltzmann Institute for Clinical Forensic Imaging and revised by all project partners. They were sent to all medical project partners via email in electronic form with input fields. In an effort to distribute the questionnaires on a European-wide basis, the questionnaires were also dispatched to about 180 relevant stakeholders, such as ministries of justice and health, medical associations, members of the European Council of Legal Medicine [19], and experts in law and forensic medicine.

## QCFN

The questionnaire comprised 32 items and was divided into three parts: Part I enquired about the current status of clinical forensic examination services; Part II covered routine clinical forensic examination practice; and Part III dealt with the expectations towards a future CFN Europe. The survey was carried out from May 2017 till January 2018. The first responses were received in July 2017 and the last responses in March 2018. Responses from 13 European countries were obtained: Austria, Croatia, Czech Republic, Germany, Greece, Ireland, Italy, Luxembourg, Poland, Portugal, Romania, Slovakia and Slovenia. The following results are based on the survey responses.

### Part I: current status of clinical forensic examination services

With the initial questionnaire items (Supplementary Material S1), the general availability of clinical forensic examination provided in each country was assessed. In brief, 12 of the 13 countries offered clinical forensic examinations (Austria, Czech Republic, Germany, Greece, Ireland, Italy, Luxembourg, Poland, Portugal, Romania, Slovakia and Slovenia). Nine of those countries offered examinations on a low-threshold basis: a person could be examined without having filed a complaint to the police regarding a criminal offence. That service was available in Austria, Germany, Ireland, Italy, Luxemburg, Poland, Portugal, Romania and Slovenia. The Czech Republic, Greece and Slovakia offered clinical forensic examinations, but not on a low-threshold basis. Those three countries considered the low-threshold service useful. In Croatia, the Institute of Forensic Medicine and Criminalistics at the University of Zagreb [20] did not offer clinical forensic examinations at the time of the study (July 2017); however, it plans to establish a clinical forensic unit in the future.

#### Service availability to victims

**Figure 1.**
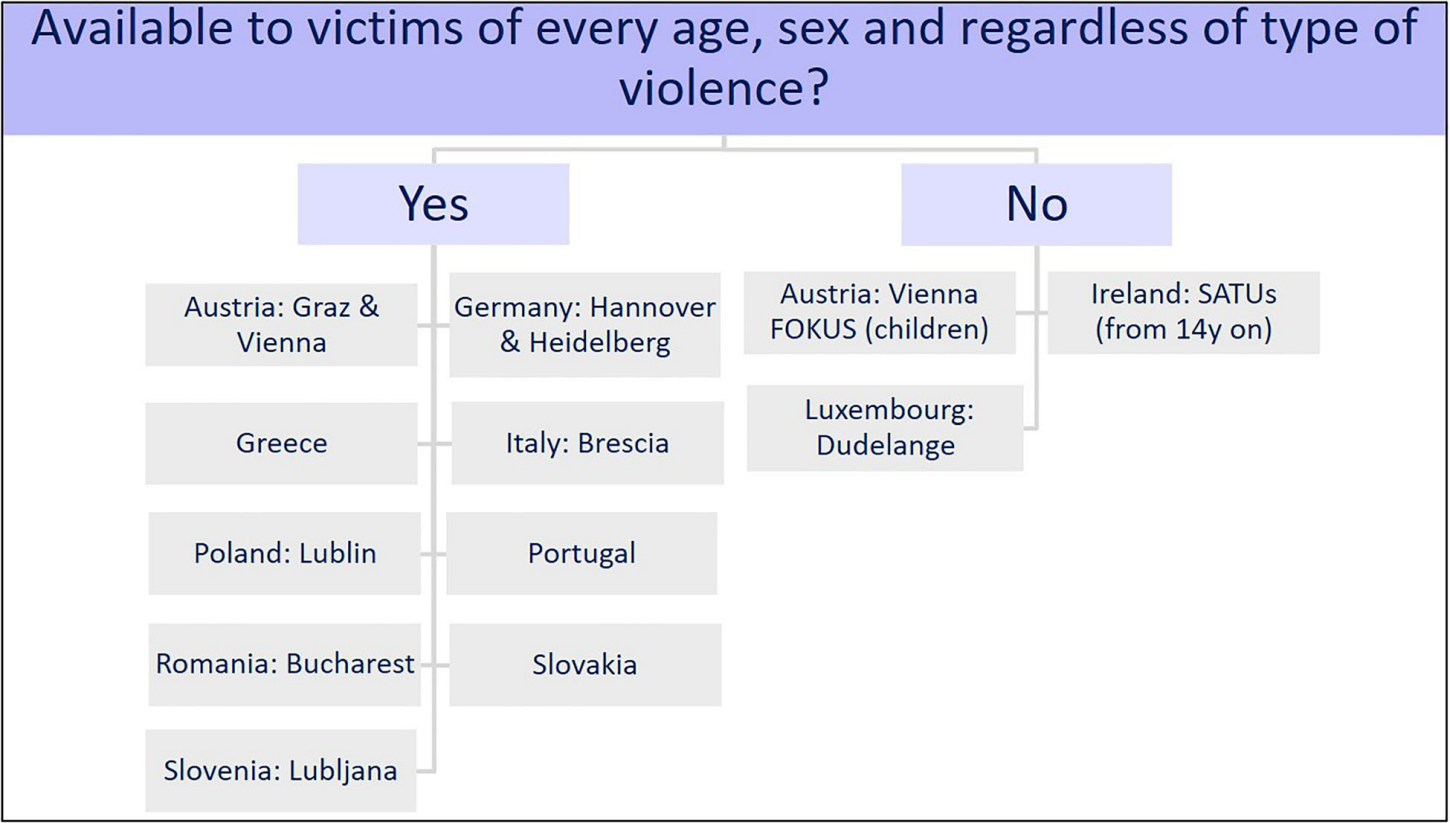
Service availability to victims. FOKUS: Forensische Kinder- und Jugenduntersuchungsstelle (in German, Forensic Outpatient Centre for children and adolescents); SATUs: sexual assault treatment units.

Another question asked whether the availability of an examination service was dependent on such factors as age, sex, or the type of violence (Supplementary Material S1). As indicated in Figure 1, nine countries answered that question in the affirmative: Austria (Graz and Vienna); Germany (Hannover and Heidelberg); Greece; Italy (Brescia); Poland (Lublin); Portugal; Romania (Bucharest); Slovakia; and Slovenia (Ljubljana). In Austria, the situation depended on the institution. There were no restrictions with the examination services in hospitals in Graz and Vienna; however, restrictions existed with an other institution in Vienna regarding the age of victims. The latter was the case for the Forensic Outpatient Centre for Children and Adolescents (FOKUS, in German: Forensische Kinder- und Jugenduntersuchungsstelle) in Vienna, which is an outpatient centre available only to children and adolescents aged up to 18 years [21]. In Lower Saxony in Germany, the Network ProBeweis (in German: Netzwerk ProBeweis) consisted of 36 hospitals and offered clinical forensic examinations only in cases of domestic violence and sexual abuse [22]. In Hannover, a special centre for the assessment regarding possible child abuse (in German: Kinderschutzambulanz) was available [23]. In Ireland, six Sexual Assault Treatment Units (SATUs) were subject to two restrictions: the units were accessible only to women and men older than 14 years and in cases of suspected sexual violence. Some services for children younger than 14 years existed in Ireland, but at the time of the questionnaire, they were dispersed over a wider geographical area and were generally not standardized [24]. In Luxembourg, it should be noted that children could not be examined on a low-threshold basis by the Unit for Medicolegal Documentation of Injuries (UMEDO) owing to reporting obligations [25]. No questionnaire response about a low-threshold service availability was received from Slovakia.

#### On-call service

The survey included questions about the availability of an on-call service and on-call hours.

**Figure 2.**
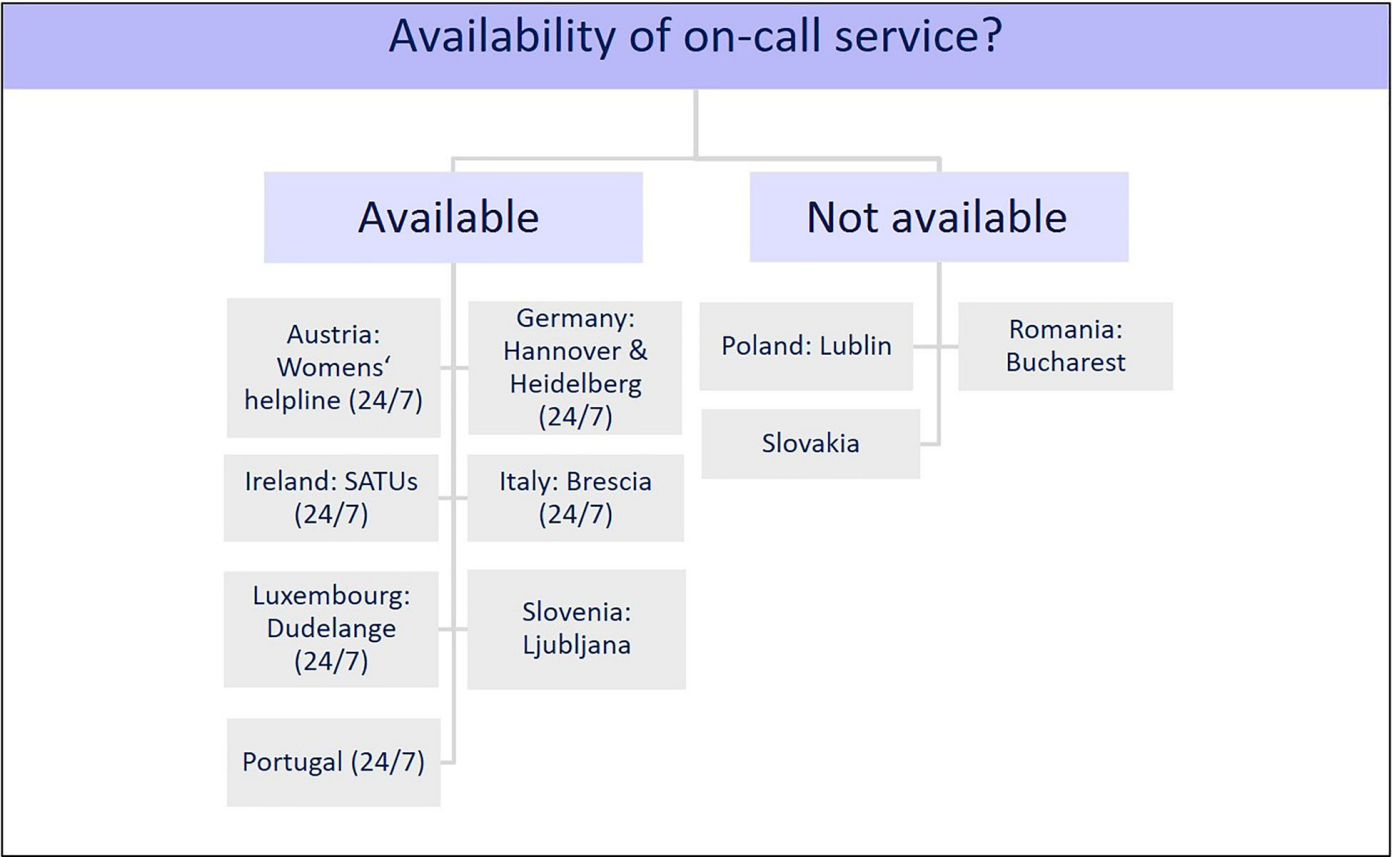
On-call service. SATUs: sexual assault treatment units.

As Figure 2 demonstrates, six European states (Austria, Germany, Ireland, Italy, Luxembourg and Portugal) provided a 24-h on-call service. In Austria, this service was provided through the Women’s helpline against violence (in German: Frauenhelpline gegen Gewalt) [26]. In Germany, the two cities offered continuous accessibility by telephone: Hannover established a hotline within Network ProBeweis; and Heidelberg offered a hotline within its Clinical Forensic Outpatient Clinic (in German: Klinisch-Forensische Ambulanz) [27]. A 24-h on-call service was available in Ireland through SATUs [24], in Luxembourg through the UMEDO [25], and in Portugal through the National Institute of Legal Medicine and Forensic Sciences (NILMFS, in Portuguese: Instituto Nacional de Medicina Legal e Ciências Forenses) [28]. In Italy, such a service was available through the Spedali Civili di Brescia, a hospital in Brescia [29]. An on-call service was not available in Lublin (Poland) [30], Bucharest (Romania) [31] and Slovakia. In Bratislava (Slovakia), an on-call service was organized on an informal base that included 11 qualified forensic pathologists. In Ljubljana (Slovenia) [32], an on-call service was available, but the on-call hours were not specified. No data about an on-call service were received from Greece and Czech Republic.

#### Provision of clinical forensic services

**Figure 3.**
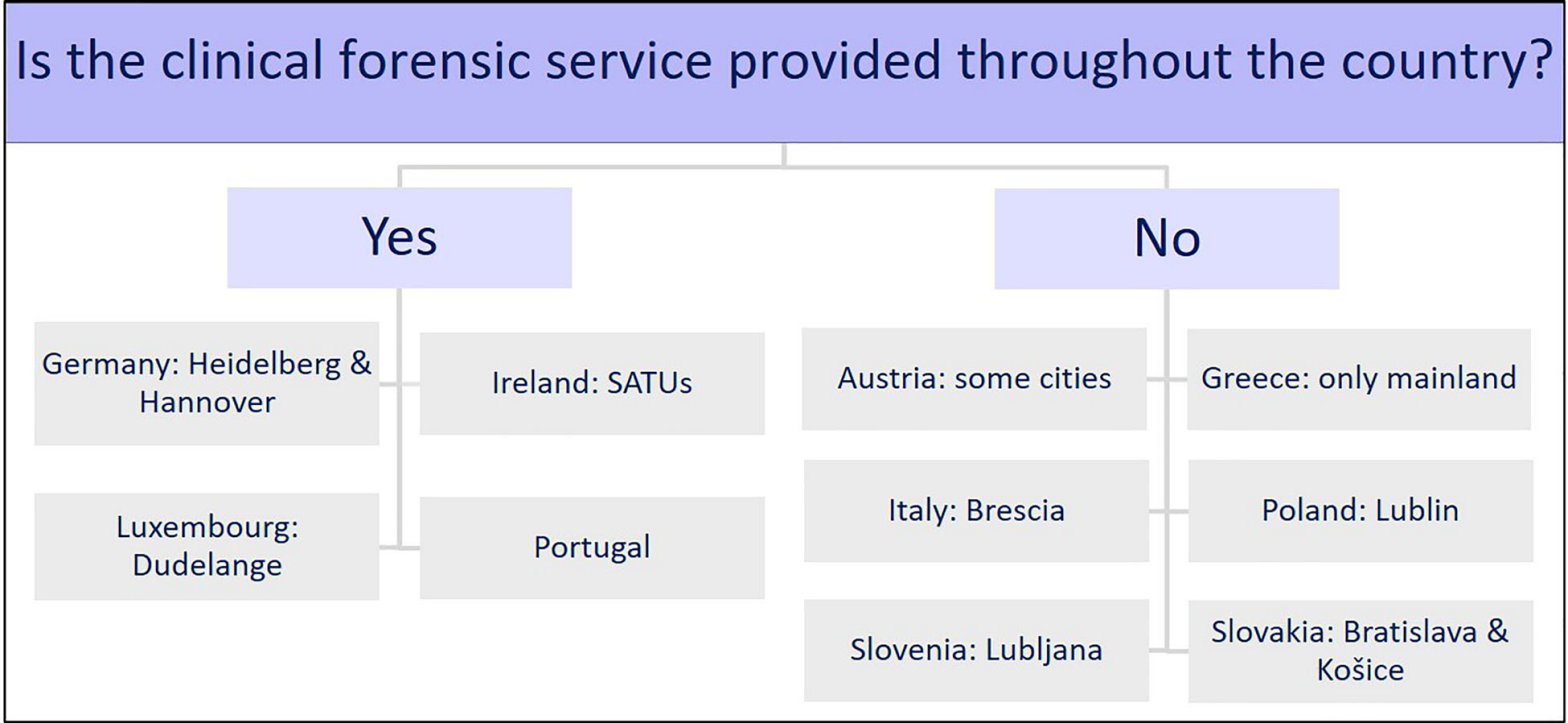
Regional service limitations. SATUs: sexual assault treatment units.

With regard to the nationwide provision of clinical forensic services (Supplementary Questionnaire S1), the responses appear in Figure 3.

With Heidelberg (Germany), the clinical forensic examinations took place within a radius of ∼200 km from the city [27]; Hannover operated the Network ProBeweis, which covers the whole state of Lower Saxony with its partner hospitals [22]. The UMEDO and its four partner hospitals provided clinical forensic examinations throughout Luxembourg. In Ireland, every person was able to reach one of the six SATUs within 3-h driving time [24]. In Portugal, the NILMFS [28] covered the whole country with its 33 service facilities.

In Austria, the service was more or less restricted to some larger cities (Graz, Innsbruck, Linz, Salzburg and Vienna) [33]. In Greece, forensic services were available only on the mainland. The forensic service through the hospital Spedali Civili di Brescia in Italy was limited to the city of Brescia and its suburbs [29]. That situation was similar for the service of the Institute of Forensic Medicine for the city of Ljubljana (Slovenia) and its suburbs [32]. Likewise in Lublin (Poland) [30] and in Bratislava [34] and Košice (Slovakia) [35], the service was provided only through the department of forensic medicine. No data about regional service limitations were received from Romania and Czech Republic.

#### Access to clinical forensic services

Regarding clinical forensic examination services, the survey also included a question about how a victim could contact a clinical forensic service facility at a low-threshold level (Supplementary Material S1). The situation varied from country to country and was sometimes not even consistent within the same country. One possibility for the victim to gain access to an examination was through self-referral via the Internet, email, or an on-call service. For example in Heidelberg (Germany), the victim could directly call the Clinical Forensic Outpatient Clinic [27]. In Ireland, the SATUs, contacted directly by a patient, offered victims a choice between a health check (e.g. providing emergency contraception and sexually transmitted infection (STI) prophylaxis) or a forensic examination (also including emergency contraception and STI prophylaxis) [24]. In Portugal, the NILMFS could be contacted directly, and it forwarded a complaint to court if the victim consented [28]. Another approach was to establish contact through the hospital emergency room, which was the routine procedure in Brescia (Italy) [29], or through partner hospitals if such a service has been established (e.g. within the Network Pro Beweis in Lower Saxony, Germany [22]). In some countries, it was possible to contact the clinical forensic service facility through victim support groups, other physicians or such authorities as the police and youth welfare authority.

#### Dissemination of clinical forensic services

In the survey, respondents made the following recommendations about further disseminating low-threshold clinical forensic examinations (Supplementary Material S1). The provided responses could be summarized in three categories: raising public awareness; political or state support; and training. However, some answers did overlap and sometimes fitted all categories. Raising public awareness related to recommendations to promote clinical forensic examinations among the general public. More coverage should be sought in the media, such as through TV and radio, as well as announcements in public bulletins and social media channels. Such moves should be accompanied by public information in the form of seminars and promotions.

The second category (political or state support) emphasized the importance of legal regulations (which would secure the funding of clinical forensic examinations on a long-term basis) as well as that of political support. A main demand made by respondents was that the reimbursement of examination costs to victims and institutions should be resolved. In general, funding should be raised for the work of physicians when dealing with victims of sexual and/or physical violence. The state and political forces should aim to increase awareness among health-care providers regarding clinical forensic examinations. Such moves could be executed by introducing official recommendations through national health authorities or legal regulations. Finally, to enhance telemedicine, a major impact could be achieved by improving access to services via the Web and phone.

The third category (training) emphasized the need for all kinds of teaching activities. Forensic training sessions should be offered for all relevant occupational groups, such as victim support groups, teachers, physicians, nursing staff, midwives and youth welfare authorities. To avoid and identify violence, improving knowledge at school plays a key role. Where networks are already established in a country, the aim should be to increase the number of partner hospitals involved in the network and expand training.

#### Examining person

**Figure 4.**
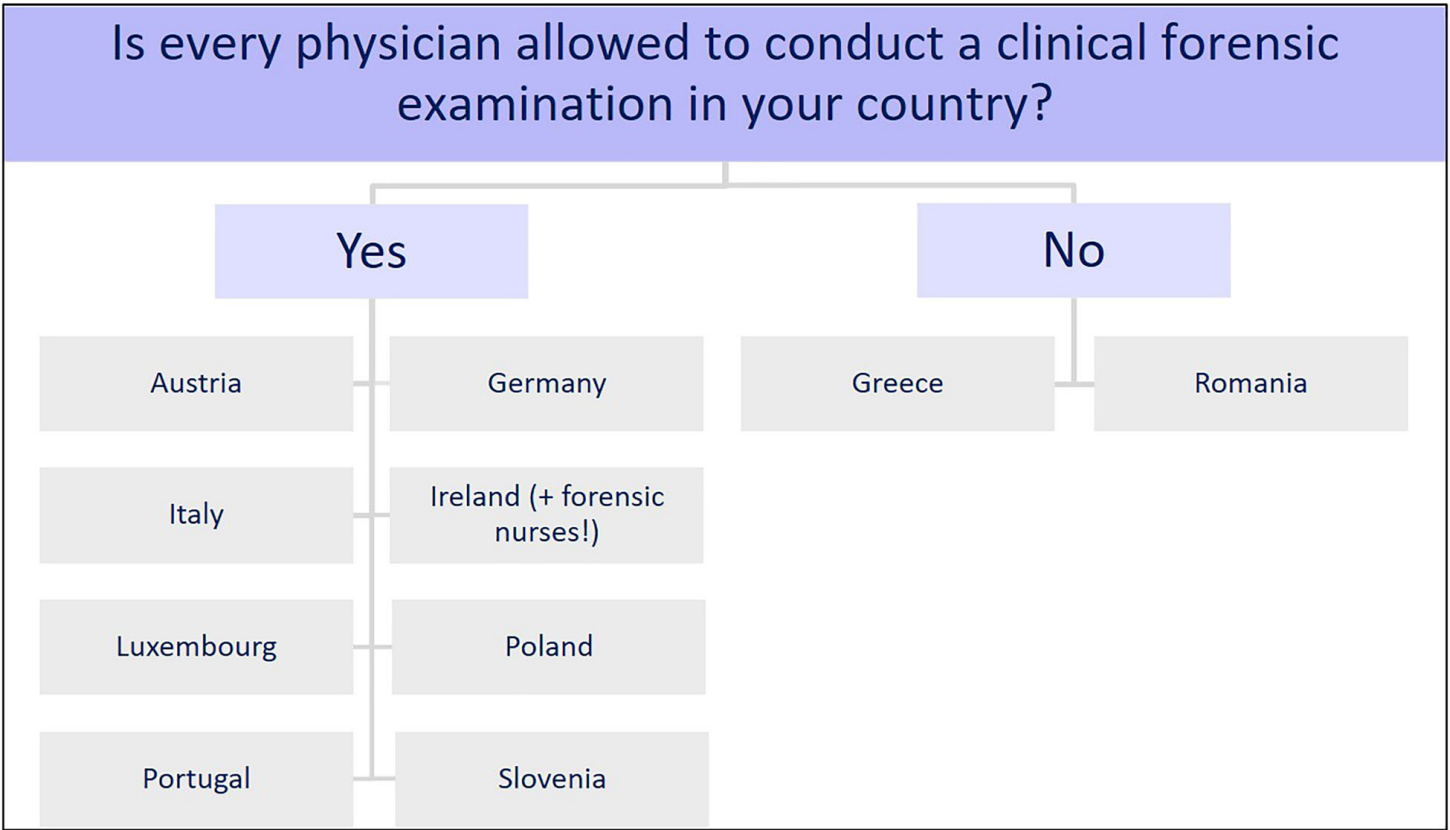
Type of examining physician.

The survey included questions about assessing the role of the person who conducts the clinical forensic examination. As Figure 4 shows, in most countries, all types of physicians (family doctors, obstetricians, paediatricians, emergency physicians, court-appointed physicians) were allowed to conduct a clinical forensic examination. These countries were Austria, Germany, Italy, Ireland, Luxembourg, Poland, Portugal and Slovenia. In Ireland, forensic nurses were trained to conduct clinical forensic examinations on men and women aged over 14 years [24]. In Greece and Romania, only a physician specialized in forensic medicine was allowed to conduct such an examination.

#### Special training for clinical forensic examinations

The questionnaire enquired whether medical staff (physicians and forensic nurses) had received special training for such examinations (Supplementary Material S1). That was answered in the affirmative for Austria, Germany, Greece, Ireland, Poland, Portugal, Slovenia and Romania. In Italy, no special training was available; thus, physicians had to rely on self-study, lectures, and seminars. Likewise in Luxembourg, no special training was available; clinical forensic examinations were performed only by board-certified forensic pathologists.

Special training for performing clinical forensic examinations has both advantages and disadvantages (Supplementary Material S1). One advantage is that forensic findings may have a higher admissibility rate as evidence in legal proceedings. Accordingly, the victims’ legal status is enhanced; the court is able to assess the case on a more objective basis, thereby promoting legal certainty [5, 36]. Another advantage is that training guarantees a higher quality standard for clinical forensic examinations and assures that the examination can be conducted in a timely manner. For example, a victim can be examined instantly by a trained physician without having to wait for a specialist to arrive. When taking into account that in rural areas no forensic physicians are usually available, it is very important that general practitioners should be empowered to perform such examinations. In addition, trained personnel are best for ensuring that a patient (or rather a victim) receives the appropriate care. Moreover, training in general increases the awareness of possible violent cases, which may have otherwise remained unnoticed, as physicians gain knowledge about identifying evidential traces and prevent their destruction. In this context, written guidelines for physicians are of great value [37, 38]. Finally, training also optimizes communication among the institutions concerned with victims of sexual and/or physical violence. The disadvantages of special training concern time and cost factors: training is rather time consuming and expensive because it needs to be undertaken regularly to ensure a consistent quality level.

### Part II: clinical forensic examination routine

Part II of the questionnaire obtained information about the clinical forensic examination routine. Of particular interest was the availability of a standardized examination kit and standardized documentation form (Supplementary Material S1). The answers varied from country to country. The SATUs in Ireland [24] and the NILMFS in Portugal [28] were best-practice examples: they provided a standardized examination kit and standardized documentation form for the whole of the country. In Austria, a standardized kit was available, which contained a standardized documentation form called MedPol–form for the examination to document injuries (in German: MedPol—Untersuchungsbogen zur Verletzungsdokumentation). The MedPol documentation form was developed by Andrea Berzlanovich within the MedPol project by the Federal Criminal Police Office of the Ministry of the Interior in cooperation with the Austrian Medical Association and Austrian Society for Forensic Medicine; it can be downloaded from the Internet [39]. The Network ProBeweis in Lower Saxony [22] and Clinical Forensic Outpatient Clinic in Heidelberg (Germany) [27] both used a standardized kit and documentation form; however, the documentation form was not standardized for the whole country. The same applied to the UMEDO in Luxembourg [25]. The Spedali Civili di Brescia in Italy [29] had a standardized kit, but it did not use a documentation form. The departments of forensic medicine in Lublin (Poland) [30] and Ljubljana (Slovenia) [32] did not have examination kits; however, each used a documentation form that was not standardized for the whole country. The National Institute of Legal Medicine Mina Minovici in Bucharest (Romania) [31] also lacked an examination kit, but it included standardized elements in the documentation form. Different regions in Romania could produce their own documentation form, which had to contain the standardized elements. No responses were obtained from Greece and Slovakia about the clinical forensic examination routine.

One interesting aspect about the course of a clinical forensic examination was photographic documentation and storage of evidence (Supplementary Material S1). At the institutions in Austria [40], Germany [22, 27], Italy [29], Luxembourg [25], Portugal [28] and Slovenia [32], photographs were routinely taken and evidentiary findings were stored. Ideally, a forensic colorimetric scale should be used when taking the images to best depict the size and colour of the injuries. The period of time for storing evidence varied among the institutions and also depended on legal regulations: it was from 6 months to 30 years. At the institutions in Ireland [24], Poland [30] and Romania [31], no photographs were taken, but evidentiary findings were stored. No data about this question were obtained from Greece and Slovakia.

### Part III: expectations towards a future CFN Europe

To conclude the survey, Part III contained questions about a future CFN Europe (Supplementary Material S1). Fortunately, all respondents from Austria, Croatia, Czech Republic, Germany, Greece, Italy, Ireland, Luxembourg, Poland, Portugal, Romania, Slovenia and Slovakia expressed their interest in joining such a network to promote the spreading of clinical forensic services in Europe.

A European-wide network could offer many advantages for victims and medical staff. Both would benefit from such a network, because it would present a strong common voice at the European level towards implementing guidelines and standards as well as funding for examination services. Further, victims would benefit by having equal rights and receive equal support and protection regardless where they are in the EU. In a clinical forensic examination, evidentiary findings have higher value if the evidence was properly obtained, which serves to strengthen the legal position of a victim in court. Another advantage is that through an interdisciplinary network, more systemic problem solving could be achieved. Moreover, through a CFN Europe, victims could access up-to-date and easily obtain information about whom to contact and where to find a specialist for a clinical forensic examination. A CFN Europe could help raise public awareness about the issue of domestic and sexual violence, which could encourage victims to come forward and report their cases. Further, a CFN Europe would encourage mutual learning among medical staff by enabling networking and research opportunities with international experts. Through such a network, experts and other medical staff could easily keep in contact, share their experiences and address urgent matters. Another advantage would be that a European network could establish standardized guidelines for examination procedures, thereby facilitating the conduct of such examinations. The network would be able to offer training for medical staff and other occupational groups in close contact with victims of physical and/or sexual violence. Through such training, medical personnel could become aware of the importance of securely and adequately storing forensic findings.

## Conclusions and outlook

The responses obtained from the QCFN questionnaire, which was developed within the JUST_e_U! project, provided a first insight into the current situation about clinical forensic services in the EU. The data from Part I of the QCFN indicated that clinical forensic examinations are of great relevance for victim support. The clinical forensic services offered were specialist support services in the sense of Article 8 Section 3 of the victims’ rights directive. Therefore, it is necessary to set the aim of further establishing or expanding such services in all European countries. To facilitate implementation of those services and based on the QCFN responses, the Ludwig Boltzmann Institute for Clinical Forensic Imaging developed a concept about expanding national clinical forensic examinations. The concept was included in the final compilation, which was forwarded to European decision makers at the end of the JUST_e_U! project in January 2019. As noted above, clinical forensic services should be built on three pillars: raising public awareness; political or state support; and training. To guarantee adequate support for victims of all forms of violence, it is important to provide an on-call service, ideally on a 24-h basis.

From the responses to Part II of the QCFN, it was evident that standardized examination kits and documentation forms would be greatly beneficial in best securing forensic findings and recording those findings. Within the JUST_e_U! project, recommendations relating to a European-wide standard for clinical forensic examinations were drafted and included in the final compilation, which can be downloaded from the JUST_e_U! homepage [41].

The responses to Part III of the QCFN revealed that there was considerable interest in a joint future CFN Europe. Such a CFN Europe could serve medical staff and victims of violence. As a first step towards establishing such a network, forensic experts from 11 European countries discussed statutes for a CFN Europe at the JUST_e_U! workshop in June 2018. The revised statutes were also included in the project’s final compilation and can be downloaded from the JUST_e_U! homepage [42]. In conclusion, it should be said that the JUST_e_U! project was a starting point for giving clinical forensic medicine a voice at the European level. Nevertheless, more data have to be collected to elaborate and improve the current situation about accessing clinical forensic examinations in the EU.

## Supplementary Material

Supplemental Material
