# Supplementary material for: Overview of clinical forensic services in various countries of the European Union
Source: Forensic Sci Res. 2019 Oct 4;5(1):74–84. doi: 10.1080/20961790.2019.1656881 (PMC7241523; doi:10.1080/20961790.2019.1656881)
Supplement: Supplemental Material [file TFSR_A_1656881_SM7367.pdf]

QCFN – questionnaire concerning national victim supporting low-threshold clinical forensic examination offers

Part I

Questions concerning clinical forensic examination services

1) Does your country or federal province offer clinical forensic examinations to victims of violence?

☐ No
☐ Yes

1a) If no to 1, please proceed with Part III "Questions concerning the Clinical Forensic Network Europe (CFN Europe)"

If yes to 1, please name all the institutions you know in your country that offer clinical forensic examinations:

2) Is only a physician allowed to conduct clinical forensic examinations in your country?

☐ No
☐ Yes

2a) If yes to 2, is only a forensic physician allowed to conduct clinical forensic examinations in your country?

☐ No
☐ Yes

2b) If no to 2, who else is allowed to conduct clinical forensic examinations (e.g. forensic nurse)?

3) Is the examination service available to victims of every age, sex, and regardless of the type of violence (meaning physical and sexualized violence)?

☐ No
☐ Yes

The project "JUSTeU! – juridical standards for clinical forensic examinations of victims of violence in Europe" is co-funded by the Justice Programme of the European Union.
JUST/2015/JACC/AG/VICT/9302

3a) **If no to 3, who can make use of the service?**

4) **Is the medical staff, who is performing clinical forensic examinations, specifically trained?**

- ☐ No
- ☐ Yes

4a) **If yes to 4, which training have they received (e.g. self-study, lectures and seminars, training course)? Please describe the training in detail (duration, providing institution/organization, contents, testing of knowledge)**

5) **In general, do you think special training for medical staff in hospitals is useful to carry out clinical forensic examinations?**

- ☐ No
- ☐ Yes

5a) **Which advantages and/or disadvantages do you personally see in specific forensic training for medical staff?**

6) **Given the case you have information about the numbers of clinical forensic examinations per institution per year, could you please indicate the respective numbers?**

Please feel free to insert also tables and graphs. We would also be interested, how many of the cases you examine are cases of sexual violence against adults, cases of physical violence against adults, cases of sexual violence against children (meaning a person under the age of 18) or cases of physical violence against children. The more data we receive the better!

INSTITUTION	NUMBERS/CASES

7) **Is it possible for a victim to be examined <u>without</u> having previously reported the case to the police (meaning to be examined on a low-threshold basis)?**

The project "JUSTeU! – juridical standards for clinical forensic examinations of victims of violence in Europe" is co-funded by the Justice Programme of the European Union.
JUST/2015/JACC/AG/VICT/9302

☐ No
☐ Yes

7a) If no to 7, would a clinical forensic examination service with a low-threshold access be desirable or useful in your opinion?

☐ No
☐ Yes

7b) If no to 7a, why not?

7c) If yes to 7, how can a victim contact a clinical forensic service facility?

Example of the Clinical-Forensic Outpatient Center: Victim support groups, physicians, police and the youth welfare authority can contact us. Additionally we offer an on-call service (the telephone number is available on the website and regularly announced in the newspaper).

8) Are the costs for the examination reimbursed, if the victim has previously <u>not</u> reported the case to the police (meaning on a low-threshold basis)?

☐ No
☐ Yes

8a) If yes to 8, who financially compensates it?

9) Are the costs for the examination reimbursed, if the victim has reported the case to the police?

☐ No
☐ Yes

9a) If yes to 9, who financially compensates it?

10) What could be done to further disseminate the concept of low-threshold clinical forensic examinations in your country?

The project "JUSTeU! – juridical standards for clinical forensic examinations of victims of violence in Europe" is co-funded by the Justice Programme of the European Union.
JUST/2015/JACC/AG/VICT/9302

11) Is an on-call service available in your country – meaning that a hospital can call a forensic physician to perform the examination, when dealing with a victim of violence?

☐ No

☐ Yes

11a) If no to 11, would an on-call service in your opinion be desirable for victims?

☐ No

☐ Yes

11b) If no to 11, would an on-call service in your opinion be desirable for physicians?

☐ No

☐ Yes

11c) If yes to 11, what are the on-call hours?

11d) If yes to 11, is the service provided for the whole country or is it geographically limited (e.g. regions, city limits)?

The project "JUSTeU! – juridical standards for clinical forensic examinations of victims of violence in Europe" is co-funded by the Justice Programme of the European Union.
JUST/2015/JACC/AG/VICT/9302

JUSTeU!

Part II

Questions concerning the clinical forensic examination routine

(only for institutions offering clinical forensic examinations)

12) How is a routine clinical forensic examination carried out by your institution? Please describe the standard procedure in a few sentences.

13) Do you have a standardized kit for the clinical forensic examination?

☐ No

☐ Yes

13a) If no to 13, would this be desirable or helpful?

☐ No

☐ Yes

13b) If yes to 13, which items does your forensic examination kit contain?

14) Are biological samples routinely collected during every clinical-forensic examination for lab testing, or only if there is a specific question?

Blood:

☐ during every clinical-forensic examination

☐ only if there is a specific question

☐ never

Urine:

☐ during every clinical-forensic examination

☐ only if there is a specific question

☐ never

Swaps:

☐ during every clinical-forensic examination

☐ only if there is a specific question

☐ never

The project "JUSTeU! – juridical standards for clinical forensic examinations of victims of violence in Europe" is co-funded by the Justice Programme of the European Union.
JUST/2015/JACC/AG/VICT/9302

……………………: (other biological samples)

☐ during every clinical-forensic examination
☐ only if there is a specific question
☐ never

……………………: (other biological samples)

☐ during every clinical-forensic examination
☐ only is there is a specific question
☐ never

15) In general, which kind of lab tests can be performed at your institution?

16) Do you routinely take photographs during a clinical forensic examination?

☐ No
☐ Yes

17) Do you use a documentation form to write down the information given by the patient/victim?

☐ No
☐ Yes

17a) If yes to 17, is the form standardized for the application in your country?

☐ No
☐ Yes

18) Do you take radiological findings, gained in a clinical context, into consideration within your expert opinion?

☐ No
☐ Yes

19) Is it possible to use imaging techniques (for example CT or MRI) to examine a person/victim if needed?

☐ No
☐ Yes

The project "JUSTeU! – juridical standards for clinical forensic examinations of victims of violence in Europe" is co-funded by the Justice Programme of the European Union.
JUST/2015/JACC/AG/VICT/9302

20) If photos and/or biological materials were collected, how long are these evidential findings stored at your institution?

☐ In low-threshold cases: ……………………
☐ In reported cases: ……………………

Part III – Questions concerning the Clinical Forensic Network Europe (CFN Europe)

21) If your country does not have a clinical forensic service yet, would you be interested in such a service?

☐ No
☐ Yes

22) Are you interested in a European wide network (CFN Europe) to strengthen the spreading of clinical forensic examination services?

☐ No
☐ Yes

23) Would you be interested to develop a European wide harmonized procedure for clinical forensic examinations?

☐ No
☐ Yes

24) Would you be interested to organize and participate in joint workshops and conferences focusing on clinical forensic medicine?

☐ No
☐ Yes

25) Would you consider it reasonable to have a CFN representative who pursues the interests of the network?

☐ No
☐ Yes

26) Do you think that exchanging experience about clinical forensic examinations on an international (European) level would be fruitful for mutual learning?

☐ No
☐ Yes

27) Would you be interested in developing and refining guidelines for clinical forensic examinations within a European-wide network?

☐ No
☐ Yes

The project "JUSTeU! – juridical standards for clinical forensic examinations of victims of violence in Europe" is co-funded by the Justice Programme of the European Union.
JUST/2015/JACC/AG/VICT/9302

28) Would you be interested in developing a standardized kit for clinical forensic examinations within a European-wide network?

☐ No

☐ Yes

29) Which advantages do you see for medical staff attending a European CFN?

30) Which advantages could a CFN offer victims of (physical, sexualized) violence?

31) Which projected problems could arise during the establishment of a CFN?

32) Do you have any further suggestions or statements about CFN Europe:

The project "JUSTeU! – juridical standards for clinical forensic examinations of victims of violence in Europe" is co-funded by the Justice Programme of the European Union.
JUST/2015/JACC/AG/VICT/9302
